# Brain Structural Alterations in Left-Behind Children: A Magnetic Resonance Imaging Study

**DOI:** 10.3389/fncir.2019.00033

**Published:** 2019-05-08

**Authors:** Yuchuan Fu, Yuan Xiao, Meimei Du, Chuanwan Mao, Gui Fu, Lili Yang, Xiaozheng Liu, John A. Sweeney, Su Lui, Zhihan Yan

**Affiliations:** ^1^Department of Radiology, The Second Affiliated Hospital and Yuying Children’s Hospital of Wenzhou Medical University, Wenzhou, China; ^2^Department of Radiology, Center for Medical Imaging, West China Hospital of Sichuan University, Chengdu, China; ^3^Department of Psychiatry and Behavioral Neuroscience, University of Cincinnati, Cincinnati, OH, United States

**Keywords:** left-behind children, gray matter volume, fractional anisotropy, MRI, cognition

## Abstract

Parental migration has caused millions of children left behind, especially in China and India. Left-behind children (LBC) have a high risk of mental disorders and may present negative life outcomes in the future. However, little is known whether there are cerebral structural alterations in LBC in relative to those with parents. This study is to explore the effect of parental migration on brain maturation by comparing gray matter volume (GMV) and fractional anisotropy (FA) of LBC with well-matched non-LBC. Thirty-eight LBC (21 boys, age = 9.60 ± 1.8 years) and 30 non-LBC (19 boys, age = 10.00 ± 1.95 years) were recruited and underwent brain scans in 3.0 T MR. Intelligence quotient and other factors including family income, guardians’ educational level and separation time were also acquired. GMV and FA were measured for each participant and compared between groups using 2-sample *t*-tests with atlas-based analysis. Compared to non-LBC, LBC exhibited greater GMV in emotional and cortico-striato-thalamo-cortical circuits, and altered FA in bilateral superior occipitofrontal fasciculi and right medial lemniscus (*p* < 0.05, Cohen’s *d* > 0.89, corrected for false-discovery rate). Other factors including family income, guardians’ educational level and separation time were not associated with these brain changes. Our study provides empirical evidence of altered brain structure in LBC compared to non-LBC, responsible for emotion regulation and processing, which may account for mental disorders and negative life outcome of LBC. Our study suggests that absence of direct biological parental care may impact children’s brain development. Therefore, public health efforts may be needed to provide additional academic and social/emotional supports to LBC when their parents migrate to seeking better economic circumstances, which has become increasingly common in developing countries.

## Introduction

In recent years, migration has become a common phenomenon because of reduction in barriers to international trade and immigration, and rapid urbanization. These trends have lured hundreds of millions of laborers away from impoverished hometowns in rural areas of developing countries to seek better economic circumstances. It is estimated that up to 80% migrant worker in China to leave away from impoverished hometown to developed countries or coastal cities, majorly southeast cities (Cheng and Sun, 2015). As a result, millions of children are left behind with friends and relatives, mostly grandparents (United Nations, 2009; Li et al., 2015). These children who stay at home with extended family members or boarding school when their parents migrate for at least 6 months are called “left-behind children” (LBC) (All-China Women’s Federation, 2013). In China alone, there are more than 61 million LBC (All-China Women’s Federation, 2013) – a population larger than California and New York combined.

Studies of LBC have shown increased levels of social anxiety and a lower quality of life (Zhao et al., 2014; Mazzucato et al., 2015). They also have increased rates of psychiatric syndromes later in life (Liu et al., 2009; Fan et al., 2010; Wang et al., 2015), particularly mood and anxiety disorders (Heim et al., 2010) and poor academic performance (Jingzhong and Lu, 2011). Accordingly, the phenomenon of LBC may represent a significant public health problem with long term consequences for society. However, there is not yet direct evidence for altered brain development in LBC in relative to non-LBC. There is evidence from previous studies of orphanages supporting lack of parents may lead to the alterations in brain, such as accelerated amygdala-mPFC development after maternal deprivation (Gee et al., 2013), widespread reductions of the cortical thickness especially in the prefrontal cortex (McLaughlin et al., 2014), smaller total gray matter volume (GMV) and larger amygdala volume (Mehta et al., 2009; Tottenham et al., 2010), and decreased fractional anisotropy (FA) in the body of the corpus callosum, the left and right external capsule and increased mean diffusivity and axial diffusivity in the right medial lemniscus (Bick et al., 2015b). The previous study also found that the length of time children experience orphanage rearing is associated with the alterations in GMV (Tottenham et al., 2010; Bick et al., 2015b). While the circumstances for orphanages were at times rather dire and extreme, it is quite difficult to answer whether such brain alterations occur in the less adverse circumstance of the millions of LBC world-wide raised by relatives rather than their biological parents who migrated for work opportunities.

Currently, magnetic resonance imaging (MRI) as a non-invasive technique has shown its value in objectively evaluating human brain *in vivo* (Lui et al., 2016). Especially, GMV reflected by voxel-based morphometry (VBM) has grown in popularity regarding the study of human brain at different age or under conditions. This automated voxel-based whole-brain analysis technique can comprehensively evaluate overall GMV differences between groups across all voxels (Ashburner and Friston, 2000; Good et al., 2001; Whitwell, 2009), preventing biases resulting from methods using liberal thresholds and region of interest (ROI) methods in neuroimaging studies. Meanwhile, Diffusion tensor imaging (DTI) is a MRI technique sensitive to the orientation of water diffusion restricted within the neuron sheath and myelination, provides measures of white-matter microstructure in the human brain. The orientation dependence of water diffusion – FA in DTI is thought to reflect anatomical features of neural fiber, such as axon caliber, fiber density, and myelination (Scholz et al., 2009). Our previous study has successfully revealed the white-matter microstructure changes of earthquake survivors (Chen et al., 2013). However, the brain of LBC individuals has not been well-characterized regarding both gray and white matter changes, which could unveil the neuropathological effects of being left-behind and expand our understanding of this group of individuals in developing countries.

Thus, the present cross-sectional study aimed to explore potential difference of brain GMV or FA of white matter between LBC and non-LBC. We hypothesized that: (1) LBC may exhibit changes of GMV in limbic-paralimbic system and prefrontal cortex which brain regions supporting emotion processing, as well as changes of FA values in related white matter tracts, and (2) that those brain differences would be related to the length of separation from parents and other factors.

## Materials and Methods

### Participants

The study was approved by the research ethics committee of Second Affiliated Hospital and Yuying Children’s Hospital of Wenzhou Medical University, and written informed consent was obtained from the participants and their guardians, before study participation. All participants attended the same local primary school in a town of southeastern China, and therefore had similar educational environments. Inclusion criteria for LBC were children who living with and taken care of by their grandparents because both of their parents had immigrated abroad for work for more than 6 months. In contrast, the non-LBC were children who living with their nuclear family throughout childhood. All subjects were evaluated by a child psychiatrist using the Chinese version of the SCID-I (Non-patient Edition) (Wang et al., 2009) to exclude any Axis I psychiatric diagnoses, and no first-degree relatives were known to have significant psychiatric illness. The exclusion criteria for both groups were as follows: (1) Neurological or psychiatric disorder; (2) Any systemic physical illness, such as hepatitis or diabetes; (3) Receiving medications known to affect brain function; (4) History of head trauma with significant loss of consciousness; (5) Premature or post-term birth; and (6) Malnutrition, mental deficiency or physical growth retardation.

Before MRI scanning, intelligence quotient was measured using the Chinese Wechsler Intelligence Scale for Children (C-WISC) (Gong and Cai, 1993) administered by an experienced child psychologist. Additionally, anxious and depression symptoms were assessed in the LBC group by Hamilton Anxiety Scale (HAMA) (Maier et al., 1988) and Hamilton Depression Scale (HAMD) (Hamilton, 1931), respectively. Originally, 76 children were recruited, of whom four children were excluded prior to MR imaging for the following reasons, i.e., two children were excluded because of fever, one child for lead poisoning and one child for precocious puberty. Another four subjects were excluded because of excessive head motion during MRI scans. Thus, 68 subjects (38 LBC vs. 30 non-LBC) were included in analyses reported below.

### Data Acquisition

High-resolution T1-weighted images were acquired using a 3.0T MRI system (Signa HDxt EXCITE, General Electric, Milwaukee) with a volumetric 3-dimensional spoiled gradient recall (SPGR) sequence (repetition time 9.2 ms, echo time 4.1 ms, flip angle 15°, slice thickness 1 mm) using an 8-channel phase array head coil. We used a field of view (FOV) of 240 mm × 240 mm, with an acquisition matrix 256 × 256 and left to right direction of phase-encoding to obtain 248 contiguous axial slices with a slice thickness of 1.0 mm and a voxel size of 0.94 mm × 0.94 mm × 1 mm.

Diffusion tensor imaging scans were acquired axially for the whole brain with TE/TR = 88.3 ms/8000 ms, *b*-value = 1000 s/mm^2^, FOV = 220 mm × 220 mm, Matrix = 128 × 128, 34 slices, slice thickness = 4 mm, spacing between slices = 4 mm. One diffusion weighted image was acquired for each of 36 diffusion gradient directions. Two volumes with no diffusion encoding (b0) in alternate phase encoding directions were used to correct non-linear distortion corrections due to magnetic field inhomogeneity.

### Image Processing

#### GMV

Image preprocessing and statistical analyses were performed with SPM8^1^ using the VBM toolbox (VBM8). First, a customized tissue probability map was generated with the Template-O-Matic (TOM8) Toolbox (Wilke et al., 2008) using the matched-pairs approach to accurately reflect the specific brain morphometry for the age and gender of the children in our study. The anterior commissure was identified for each image and uniformly aligned for subsequent spatial normalization of native images that were segmented into gray matter, white matter and cerebrospinal fluid (CSF) according to the unified segmentation model. Then, the re-obtained gray matter images were subjected to Jacobian modulation (volume modulation) and smoothed with a 6 mm full-width at half-maximum Gaussian kernel.

#### DTI

The data were processed using the PANDA pipeline tool^2^ for preprocessing and producing diffusion metrics. The preprocess steps were as follows: (1) data were converted from “DICOM” format to a “NIFTI” file; (2) Creation of brain mask, cropping raw images, correcting for eddy-current effects; (3) Calculation of diffusion tensor metrics. Automated atlas-based ROI analysis was used to identify differences of FA between the LBC and non-LBC groups. FSL software (FMRIB Software Library, FMRIB, Oxford, United Kingdom) was used to normalize FA images into MNI space and calculate regional diffusion metrics by averaging the values within each region of the ICBM DTI-81 atlas. Mean FA of all available white matter tracts was extracted and fed into SPSS for further data analysis.

### Statistical Analysis

#### GMV

The 2-sample *t*-tests and chi-square test were used to compare the demographic data. Global brain volume was extracted and compared between groups. Then, voxel-wise comparisons of GMV were performed between groups using 2-sample *t*-tests with age, gender and global brain volume as covariates. To control for multiple comparisons, all *t*-values comparing voxel-wise data were evaluated for significance using a threshold of *p* < 0.05 with false discovery rate (FDR) correction for multiple comparison.

#### FA

Independent sample *t*-tests was performed to compare the mean FA of all the 48 fibers within the brain between the two groups (thresholded as *p* < 0.05 after FDR correction) using age and gender as covariates (Mori and van Zijl, 1995; Bick et al., 2015b).

Then, GMV or FA in each region or fiber with significant differences between LBC and non-LBC was extracted for each subject. Correlation was performed between duration of each LBC’s separation from parents and brain structure.

## Results

### Demographic Data

Sixty-eight subjects were included in the statistical analyses for the study, including 38 subjects in the LBC group (21 boys, mean age = 9.60 ± 1.8 years, age range: 7–13 years; mean separation time = 7.00 ± 2.17 years, range: 2–11 years; mean age of separation = 22.53 ± 28.32 months, range: 1–84 months) and 30 subjects in the non-LBC group (19 boys, mean age = 10.00 ± 1.95 years, age range: 7–14 years). All subjects were right-handed. Demographic data, such as age, gender, IQ, weight, height, academic scores, special interests (such as singing, dancing as well as football), birth weight, delivery method, and annual family income did not differ between the two groups (*p* > 0.05; Table 1) except for the educational levels of the primary care givers, which was significantly lower in LBC relative to the non-LBC group (*p* < 0.05). Besides, only three LBC showed mild anxiety (HAMA score range 1~3), no LBC presented depression symptom (see Table 1).

**Table 1:** Demographic characteristics of left-behind children (LBC) and parentally reared children (non-LBC).

	Group				
	LBC		Non-LBC		
Characteristic	Mean	*SD*	Mean	SD	*p*
Age (years)	9.60	1.80	10.00	1.95	0.40
IQ-verbal	89.63	12.86	93.67	15.74	0.25
IQ-performance	97.39	13.14	99.00	14.53	0.64
IQ-full scale	92.76	13.05	95.67	14.70	0.39
Birth weight (Kg)	3.37	0.47	3.41	0.46	0.78
Height (cm)	140.34	12.18	139.53	12.80	0.79
Weight (kg)	35.85	9.88	35.62	12.51	0.93
Family income (10000 yuan/year)	16.50	9.96	15.33	9.09	0.62
Education of primary care givers (years)	4.26	2.45	7.92	3.26	0.01
Separation time (years)	7.00	2.17			
Age at parental departure (months)	22.53	28.32			
Reunion time (days/year)	26.7	16.4			
Communication time (minutes/week)	24.3	25.4			
HAMA	0.3 (range 0~3)	0.8	-	-	
HAMD	0	0	-	-	
	
	***N***	**%**	***N***	**%**	***p***
	
Gender					
Girl	17	44.7	11	36.7	0.50
Boy	21	55.3	19	63.3	0.50
Delivery					
Labor	35	92.10	23	76.70	0.15
Cesarean	3	7.90	7	23.30	0.15
Special interest					
Singing/dancing	20	52.60	16	53.30	0.95
Football/Soccer	18	47.40	14	46.70	0.95

*LBC, left-behind children; IQ, intelligence quotient; SD, standard deviation.*

### Altered GMV in LBC

**FIGURE 1:**
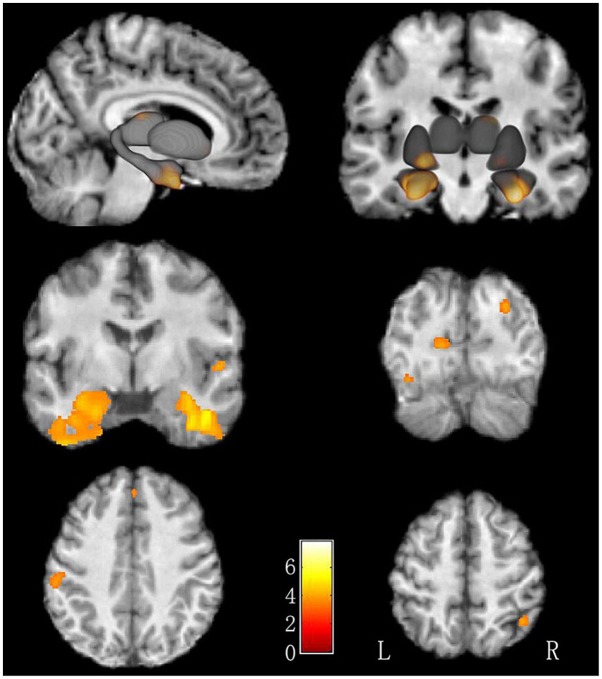
Increased GMV in LBC relative to non-LBC. Increased gray matter volume was observed in the following regions (labeled in red-yellow): bilateral fusiform gyri, bilateral parahippocampal gyri, right superior parietal lobe, right thalamus, right superior occipital gyrus, left cuneus, right superior temporal gyrus, right medial prefrontal gyrus, left postcentral gyrus, left middle occipital gyrus, and left putamen in LBC relative to non-LBC.

There was no difference of global brain volume between the two groups (*p* = 0.30). Compared to non-LBC, LBC showed significantly greater GMV in the bilateral fusiform gyri, bilateral parahippocampal gyri, right superior parietal lobe, right thalamus, right superior occipital gyrus, left cuneus, right superior temporal gyrus, right medial prefrontal cortex, left postcentral gyrus, left middle occipital gyrus and left putamen (Figure 1 and Table 2, with Cohen’s *d* > 0.89). The clusters in parahippocampi were contiguous with those in the amygdala.

**Table 2:** Voxel-based analysis of gray matter volume (GMV) in LBC relative to non-LBC study participants.

	Cluster	Peak	*P*-value	Cohen’s	Talairach coordinates
Cluster	Size	*t*-Value	(FDR-cor)	*d*	x, y, z (mm)
Fusiform_R	848	5.26	0.015	1.29	45, -5, -30
Parahippocampus_R	575	4.95	0.015	1.22	23, -36, -9
Parahippocampus_L	872	4.86	0.015	1.20	-20, -42, -12
Parietal_Sup_R	137	4.58	0.015	1.13	42, -52, 57
Fusiform_L	601	4.33	0.015	1.07	-47, -57, -20
Thalamus_R	78	4.00	0.015	0.98	18, -22, 16
Occipital_Sup_R	57	4.00	0.015	0.98	26, -81, 27
Cuneus_L	88	3.94	0.015	0.97	-15, -81, 4
Temporal_Sup_R	56	3.86	0.016	0.95	54, -4, -0
Prefrontal_medial_R	107	3.86	0.016	0.95	2, 39, 42
Postcentral gyrus_L	121	3.77	0.016	0.93	-56, -28, 37
Middle occipital Gyrus_L	62	3.75	0.016	0.92	-35, -79, -17
Putamen_L	51	3.63	0.018	0.89	-21, 18, -8

*GMV, gray matter volume; L, left; R, right; Sup, superior; FDR-cor, corrected for multiple comparisons with false discovery rate.*

### Altered FA in LBC

**FIGURE 2:**
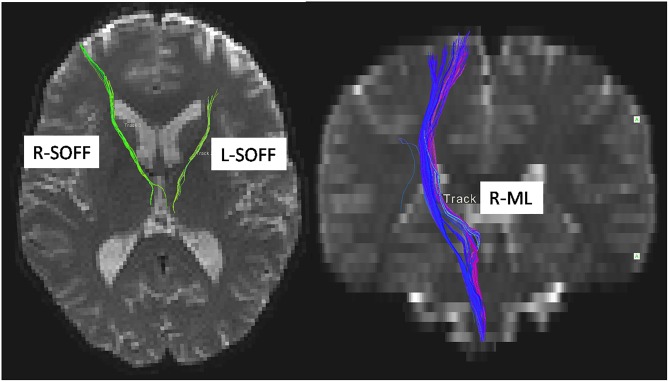
Difference of white matter microstructure between LBC and non-LBC. Tracts with significantly increased mean fractional anisotropy (FA) in the left and right superior occipitofrontal fasciculi (labeled by green color) and decreased FA in right medial lemniscus (labeled by blue color) in LBC relative to parentally raised children. Age and gender were used as covariates. L, left; R, right; ML, medial lemniscus; SOFF, superior occipitofrontal fasciculus.

We used atlas-based ROI analysis to compare the mean FA values of all 48 fiber tracts between the LBC and the non-LBC groups. When controlling for age and gender as covariates, mean FA was increased in the left and right superior occipitofrontal fasciculi and decreased in right medial lemniscus in the LBC group (Figure 2 and Supplementary Table 1).

### Relationship of Rearing Environment With Brain Structure Measures

No significant correlation was observed between the guardian’s educational level and family income and alterations of GMV in LBC.

### Relationships of Time of Reunions, Time for Telephone Communication, and Length of Separation From Parents and the Brain Structure

Some LBC children had never even met their parents as they grew up. Other children had reunions (26.7 ± 16.4 days/year; max 60 days/year). They communicated with their parents abroad via telephone or internet audiovisual software (mean time = 24.3 ± 25.4 min/week; ranges = 0–70 min/week). No significant correlations between these parental contact parameters and GMV or FA were seen in LBC. To examine effects of early separation, comparison (using age and gender as covariates) of LBC subjects with parental separation before (25 cases) and after (13 cases) 1 year of age was conducted. There was no difference in GMV or FA between these subgroups of LBC (*p* > 0.05). Children separated from parents before (29 cases) and after (9 cases) 2 years of age also did not show difference (*p* > 0.05).

## Discussion

In this study, LBC demonstrated significantly greater GMV in limbic-paralimbic and other brain regions involved in emotion regulation and processing including parahippocampal gyri, amygdala, and medial prefrontal cortex (Cardinal et al., 2002; Lui et al., 2013). These processing have been reported to be disregulated in LBC (Liu et al., 2009; Fan et al., 2010; Wang et al., 2015). Other regions with group differences belong to cortico-striato-thalamo-cortical circuits, which are crucial for cognition (Metzger et al., 2013). Analysis of white matter tracts revealed mean FA value in the bilateral superior occipitofrontal fasciculi in LBC was increased when compared with non-LBC, but decreased in the right medial lemniscus. Thus, our findings provide empirical evidence in supporting of the hypothesis that direct biological parental rearing, relative to rearing by grandparents may affect brain development of children. Furthermore, these brain changes involving emotion circuit may represent antecedent alterations in brain development that contribute to the increased risk for psychiatric disorders in later life seen in LBC (McLaughlin et al., 2014). We also found no correlation between the time of reunion, time for telephone communication, or duration of separation from parents with GMV and FA, and no significant difference in GMV and FA between the subgroups of LBC with a parental departure before or after 1 or 2 years of age.

The previous studies found that the developmental trajectory of the normal brain GMV was inverted U-shaped trajectories, and the developmental curves for the frontal and parietal lobe peaking were at about age 12 and for the temporal lobe at about age 16 (Giedd et al., 1999; Gogtay et al., 2004). However, all subjects in our study were under the 12 years old, the “hyper-structural” pattern of GMV in LBC was interpreted to be “over-maturation.” The main reason may be that the LBC couldn’t gain the comfort and guidance from their parents in the process of self-development and socialization due to the long-term parental separation, it caused them have higher loneliness feeling and social anxiety, increased life stress (Zhao et al., 2014; Mazzucato et al., 2015; Wang et al., 2015). Stress activates the Limbic-Hypothalamic-Pituitary-Adrenal Axis and elevates levels of cortisol. Cortisol regulates the stress response system both in the hippocampus and medial prefrontal cortex, where it attenuates the stress response, and in the amygdala, where it promotes that response (Bellis and Zisk, 2014). Of note, larger amygdala volumes seen in our study have also been reported in orphans (Tottenham et al., 2010). Second, greater GMV in sensory, limbic-paralimbic and emotional-regulatory systems may result from increased dendritic branching, dendritic length and spine density (Muhammad and Kolb, 2011) or from neurogenesis (Kaplan, 2001). Such effects have been reported in studies of mammals and humans exposed to early-life maternal separation (Kaplan, 2001; Muhammad and Kolb, 2011).

In contrast to the results seen in the present study, previous study of children reared in public institutions showed reductions in cortical thickness and smaller the total GMV, decreased FA of superior occipitofrontal fasciculus (Mehta et al., 2009; McLaughlin et al., 2014; Bick et al., 2015b; Yang et al., 2015), and prolonged the length of time children experience orphanage rearing was associated with larger amygdala volume and reduced microstructural integrity of the body of the corpus callosum and tracts involved in limbic circuitry, and sensory processing (Tottenham et al., 2010; Bick et al., 2015b). Several possible reasons may explain these different observations. First, the living environment of children reared in institutions in some prior studies of orphans was quite extreme compared to those of LBC. The LBC are mostly taken care of by their grandparents and have relatively intact care giving support. What a more, in a typical Chinese culture, grandparents who are seeing their grandchildren as “only family treasure” express their love via indulge their grandchildren such as providing better physiological demands and protecting them from taking parts in household chores (Kelley et al., 2011; Williams, 2011). However, many of those institutionalized children grew up in a stunningly unstimulating and unresponsive environment (Marshall, 2014), which has been believed to cause dysmaturational effects resulting in regional reduction of gray matter (Mehta et al., 2009). In comparison to the institutionalized children, LBC typically lived in a more “open” and socially stimulating environment. Second, while the possibility that more modest socioemotional deprivation may lead to the opposite pattern of brain changes than is seen in more severe deprivation is most interesting.

The prior studies showed that decreased FA of superior occipitofrontal fasciculus was associated with spatial neglect in humans (Karnath et al., 2009; Yang et al., 2015). While the higher FA of superior occipitofrontal fasciculus seen in LBC may reflect increased myelination and neuronal remodeling (Beaulieu, 2002; Li et al., 2011) at a more molecular level, it is possible that a hyperattentiveness to the visual environment related to feelings of separation and concern about available social supports might be one factor leading to the overdevelopment of this tract. Though we did not test spatial abilities in this study, the evidence from prior rodent study has shown that spatial learning was improved after early life maternal deprivation (Loi et al., 2014). The current study also demonstrated decreased FA in the medial lemniscus in LBC. This is consistent with findings in institutionalized children (Bick et al., 2015a) as well as children neglected in early life (Hanson et al., 2013), which might result from insufficient sensory input experienced at critical points in neural development owing to reduction in maternal touch and other sensory stimulation. Previous studies pointed that, maternal touch had a positive relationship with the brain development, especially in the social brain (McGlone et al., 2014; Brauer et al., 2016), of which the medial lemniscus is a crucially relevant afferent pathway. Thus, the reduced integrity of the medial lemniscus in LBC observed here provides further evidence to support the role of parental care in brain development of brain, especially systems relevant for social and emotional processing.

The present study showed that no LBC suffered from obviously anxious or depression symptom, which suggests that the brain structural alteration may precede the occurrence of clinical symptom. The finding is consistent with a prior study (Luby et al., 2013). However, because of the limited sample size of the present study, more researches with a large sample size are needed. Additionally, longitudinal design is needed to explore such dynamic changes of brain development in LBC. While findings from our cross-sectional study are promising, it is possible that genetics or other factors in parents who decides to leave families for distant opportunities might be associated themselves with varying patterns of brain development. Longitudinal studies would contribute to resolving this possibility as well. Longitudinal studies might also clarify the reversibility of brain changes, as would studies examining children after social enhancement programs were made available to LBC. Second, more extensive evaluation of behavioral, emotional, cognitive, and social development in future studies is needed to clarify the neurobehavioral significance of neuroanatomic changes seen in LBC. Nonetheless, while many questions remain to be answered, findings from our cross-sectional study of neuroanatomic differences in LBC relative to parentally raised children raised a concern that brain maturation may be altered less severe deprivation conditions than have been previously studied, and therefore in the many millions of children left by parents in the developing world to pursue better work opportunities.

## Conclusion

Despite the limitations of this work, our study provides empirical evidence of altered cerebral structure in LBC, suggesting that absence of direct biological parental care may have a negative impact on children’s brain development. From a public health perspective, our MRI study highlights the potential importance of limited parental rearing in LBC, which is known to have adverse cognitive and psychiatric sequelae. Thus, programs providing more emotional care and stimulation are needed for LBC in developing countries to reduce potential adverse long-term consequences on the individual children and for overall population health.

## Ethics Statement

The study was approved by the research ethics committee of Second Affiliated Hospital and Yuying Children’s Hospital of Wenzhou Medical University, and written consent was obtained from guardians and assent from children before study participation.

## Author Contributions

SL and ZY conceived the study and designed the protocol. YF, YX, LY, ZY, MD, SL, and CM did the experiments. YF, YX, GF, LY, and XL conducted the statistical analyses. JS, ZY, and SL interpreted the study findings and contributed to developing the manuscript. YF, YX, GF, and LY wrote the first draft of the manuscript that was revised by all authors.

## Conflict of Interest Statement

The authors declare that the research was conducted in the absence of any commercial or financial relationships that could be construed as a potential conflict of interest.

## Footnotes

1 www.fil.ion.ucl.ac.uk/spm

2 http://www.nitrc.org/projects/panda/

## References

[B1] All-China Women’s Federation (2013). *China Women’s Federation. National Survey of Left-Behind Children in Rural areas and Migrant Children in Urban and Rural Areas.* Available at: http://acwf.people.com.cn/n/2013/0510/c99013-21437965.html (accessed on 6 5, 2013).

[B2] Ashburner J. Friston K. J. (2000). Voxel-based morphometry–the methods. *Neuroimage* 11 805–821. 10.1006/nimg.2000.0582 10860804

[B3] Beaulieu C. (2002). The basis of anisotropic water diffusion in the nervous system - a technical review. *NMR Biomed.* 15 435–455. 10.1002/nbm.782 12489094

[B4] Bellis M. D. D. Zisk A. (2014). The biological effects of childhood trauma. *Child Adolesc. Psychiatr. Clin. N. Am.* 23 185–222. 10.1016/j.chc.2014.01.002 24656576 PMC3968319

[B5] Bick J. Fox N. Zeanah C. Nelson C. A. (2015a). Early deprivation, atypical brain development, and internalizing symptoms in late childhood. *Neuroscience* 342 845–853. 10.1016/j.neuroscience.2015.09.026 26384960 PMC4794419

[B6] Bick J. Zhu T. Stamoulis C. Fox N. A. Zeanah C. Nelson C. A. (2015b). Effect of early institutionalization and foster care on long-term white matter development: a randomized clinical trial. *JAMA Pediatr.* 169 211–219. 10.1001/jamapediatrics.2014.3212 25622303 PMC4413892

[B7] Brauer J. Xiao Y. Poulain T. Friederici A. D. Schirmer A. (2016). Frequency of maternal touch predicts resting activity and connectivity of the developing social brain. *Cereb. Cortex* 26 3544–3552. 10.1093/cercor/bhw137 27230216 PMC4961023

[B8] Cardinal R. N. Parkinson J. A. Hall J. Everitt B. J. (2002). Emotion and motivation: the role of the amygdala, ventral striatum, and prefrontal cortex. *Neurosci. Biobehav. Rev.* 26 321–352. 10.1016/S0149-7634(02)00007-6 12034134

[B9] Chen L. Lui S. Wu Q. Z. Zhang W. Zhou D. Chen H. F. (2013). Impact of acute stress on human brain microstructure: an MR diffusion study of earthquake survivors. *Hum. Brain Mapp.* 34 367–373. 10.1002/hbm.21438 22042533 PMC6870418

[B10] Cheng J. Sun Y. H. (2015). Depression and anxiety among left-behind children in china: a systematic review. *Child Care Health Dev.* 41 515–523. 10.1111/cch.12221 25495395

[B11] Fan F. Su L. F. Gill M. K. Birmaher B. (2010). Emotional and behavioral problems of Chinese left-behind children: a preliminary study. *Soc. Psychiatry Psychiatr. Epidemiol.* 45 655–664. 10.1007/s00127-009-0107-4 19657573

[B12] Gee D. G. Gabard-Durnam L. J. Flannery J. Goff B. Humphreys K. L. Telzer E. H. (2013). Early developmental emergence of human amygdala-prefrontal connectivity after maternal deprivation. *Proc. Natl. Acad. Sci. U.S.A.* 110 15638–15643. 10.1073/pnas.1307893110 24019460 PMC3785723

[B13] Giedd J. N. Blumenthal J. Jeffries N. O. Castellanos F. X. Rapoport J. L. (1999). Brain development during childhood and adolescence: a longitudinal mri study. *Nat. Neurosci.* 2 861–863. 10.1038/13158 10491603

[B14] Gogtay N. Giedd J. N. Lusk L. Hayashi K. M. Greenstein D. Vaituzis A. C. (2004). Dynamic mapping of human cortical development during childhood through early adulthood. *PNAS* 101 8174–8179. 10.1073/pnas.0402680101 15148381 PMC419576

[B15] Gong Y. Cai T. (1993). *Manual of Chinese Revised Wechsler intelligence scale for children.* Changsha: Hunan Atlas Publishing House.

[B16] Good C. D. Johnsrude I. S. Ashburner J. Henson R. Friston K. J. Frackowiak R. S. (2001). A voxel-based morphometric study of ageing in 465 normal adult human brains. *Neuroimage* 14 21–36. 10.1109/SSBI.2002.1233974 11525331

[B17] Hamilton M. (1931). *Hamilton Depression Scale.* Zurich: Psychiatric University Hospital Zurich.

[B18] Hanson J. L. Adluru N. Chung M. K. Alexander A. L. Davidson R. J. Pollak S. D. (2013). Early neglect is associated with alterations in white matter integrity and cognitive functioning. *Child Dev.* 84 1566–1578. 10.1111/cdev.12069 23480812 PMC3690164

[B19] Heim C. Shugart M. Craighead W. E. Nemeroff C. B. (2010). Neurobiological and psychiatric consequences of child abuse and neglect. *Dev. Psychobiol.* 52 671–690. 10.1002/dev.20494 20882586

[B20] Jingzhong Y. Lu P. (2011). Differentiated childhoods: impacts of rural labor migration on left-behind children in China. *J. Peasant Stud.* 38 355–377. 10.1080/03066150.2011.559012 21744548

[B21] Kaplan M. S. (2001). Environment complexity stimulates visual cortex neurogenesis: death of a dogma and a research career. *Trends Neurosci.* 24 617–620. 10.1016/S0166-2236(00)01967-6 11576677

[B22] Karnath H. O. Rorden C. Ticini L. F. (2009). Damage to white matter fiber tracts in acute spatial neglect. *Cereb. Cortex* 19 2331–2337. 10.1093/cercor/bhn250 19168667 PMC2742593

[B23] Kelley S. J. Whitley D. M. Campos P. E. (2011). Behavior problems in children raised by grandmothers: the role of caregiver distress, family resources, and the home environment. *Child Youth Serv. Rev.* 33 2138–2145. 10.1016/j.childyouth.2011.06.021

[B24] Li F. Huang X. Yang Y. Li B. Wu Q. Zhang T. (2011). Microstructural brain abnormalities in patients with obsessive-compulsive disorder: diffusion-tensor MR imaging study at 3.0 T. *Radiology* 260 216–223. 10.1148/radiol.11101971 21474704

[B25] Li Q. Liu G. Zang W. (2015). The health of left-behind children in rural China. *China Econ. Rev.* 36 367–376. 10.1016/j.chieco.2015.04.004

[B26] Liu Z. Li X. Ge X. (2009). Left too early: the effects of age at separation from parents on Chinese rural children’s symptoms of anxiety and depression. *Am. J. Public Health* 99 2049–2054. 10.2105/AJPH.2008.150474 19762669 PMC2759782

[B27] Loi M. Koricka S. Lucassen P. J. Joels M. (2014). Age- and sex-dependent effects of early life stress on hippocampal neurogenesis. *Front. Endocrinol.* 5:13 10.3389/fendo.2014.00013 PMC3929839 24600436

[B28] Luby J. Belden A. Botteron K. Marrus N. Harms M. P. Babb C. (2013). The effects of poverty on childhood brain development: the mediating effect of caregiving and stressful life events. *JAMA Pediatr.* 167 1135–1142. 10.1001/jamapediatrics.2013.3139 24165922 PMC4001721

[B29] Lui S. Chen L. Yao L. Xiao Y. Wu Q. Z. Zhang J. R. (2013). Brain structural plasticity in survivors of a major earthquake. *J. Psychiatry Neurosci.* 38 381–387. 10.1503/jpn.120244 23710694 PMC3819151

[B30] Lui S. Zhou X. J. Sweeney J. A. Gong Q. (2016). Psychoradiology: the frontier of neuroimaging in psychiatry. *Radiology* 281 357–372. 10.1148/radiol.2016152149 27755933 PMC5084981

[B31] Maier W. Buller R. Philipp M. Heuser I. (1988). The Hamilton anxiety scale: reliability, validity and sensitivity to change in anxiety and depressive disorders. *J. Affect. Disord.* 14 61–68. 10.1016/0165-0327(88)90072-9 2963053

[B32] Marshall E. (2014). An experiment in zero parenting. *Science* 345 752–754. 10.1126/science.345.6198.752 25124426

[B33] Mazzucato V. Cebotari V. Veale A. White A. Grassi M. Vivet J. (2015). International parental migration and the psychological well-being of children in Ghana, Nigeria, and Angola. *Soc. Sci. Med.* 132 215–224. 10.1016/j.socscimed.2014.10.058 25464874

[B34] McGlone F. Wessberg J. Olausson H. (2014). Discriminative and affective touch: sensing and feeling. *Neuron* 82 737–755. 10.1016/j.neuron.2014.05.001 24853935

[B35] McLaughlin K. A. Sheridan M. A. Winter W. Fox N. A. Zeanah C. H. Nelson C. A. (2014). Widespread reductions in cortical thickness following severe early-life deprivation: a neurodevelopmental pathway to attention-deficit/hyperactivity disorder. *Biol. Psychiatry* 76 629–638. 10.1016/j.biopsych.2013.08.016 24090797 PMC3969891

[B36] Mehta M. A. Golembo N. I. Nosarti C. Colvert E. Mota A. Williams S. C. R. (2009). Amygdala, hippocampal and corpus callosum size following severe early institutional deprivation: the english and romanian adoptees study pilot. *J. Child Psychol. Psychiatry* 50 943–951. 10.1111/j.1469-7610.2009.02084.x 19457047

[B37] Metzger C. D. Vand W. Y. D. Walter M. (2013). Functional mapping of thalamic nuclei and their integration into cortico-striatal-thalamo-cortical loops via ultra-high resolution imaging—from animal anatomy to in vivo imaging in humans. *Front. Neurosci.* 7:24. 10.3389/fnins.2013.00024 23658535 PMC3647142

[B38] Mori S. van Zijl PC (1995). Diffusion weighting by the trace of the diffusion tensor within a single scan. *Magn. Reson. Med.* 33 41–52. 10.1002/mrm.1910330107 7891534

[B39] Muhammad A. Kolb B. (2011). Maternal separation altered behavior and neuronal spine density without influencing amphetamine sensitization. *Behav. Brain Res.* 223 7–16. 10.1016/j.bbr.2011.04.015 21515311

[B40] Scholz J. Klein M. C. Behrens T. E. Johansen-Berg H. (2009). Training induces changes in white-matter architecture. *Nat. Neurosci.* 12 1370–1371. 10.1038/nn.2412 19820707 PMC2770457

[B41] Tottenham N. Hare T. A. Quinn B. T. McCarry T. W. Nurse M. Gilhooly T. (2010). Prolonged institutional rearing is associated with atypically large amygdala volume and difficulties in emotion regulation. *Dev. Sci.* 13 46–61. 10.1111/j.1467-7687.2009.00852.x 20121862 PMC2817950

[B42] United Nations (2009). *International Migration.* New York, NY: United Nations.

[B43] Wang D. Yang S. Jiang C. Michael P. (2009). *Structured Clinical Interview for DSM-IV-TR Axis I Disorders, Research Version, Chinese Revision.* Beijing: Beijing Suicide Research and Prevention Center.

[B44] Wang X. Ling L. Su H. Cheng J. Jin L. Sun Y. H. (2015). Self-concept of left-behind children in China: a systematic review of the literature. *Child Care Health Dev.* 41 346–355. 10.1111/cch.12172 25039693

[B45] Whitwell J. L. (2009). Voxel-based morphometry: an automated technique for assessing structural changes in the brain. *J. Neurosci.* 29 9661–9664. 10.1523/jneurosci.2160-09.2009 19657018 PMC6666603

[B46] Wilke M. Holland S. K. Altaye M. Gaser C. (2008). Template-O-Matic: a toolbox for creating customized pediatric templates. *Neuroimage* 41 903–913. 10.1016/j.neuroimage.2008.02.056 18424084

[B47] Williams M. N. (2011). The changing roles of grandparents raising grandchildren. *J. Hum. Behav. Soc. Environ.* 21 948–962. 10.1080/10911359.2011.588535 10776166

[B48] Yang W. Liu T. T. Song X. B. Zhang Y. Li Z. H. Cui Z. H. (2015). Comparison of different stimulation parameters of repetitive transcranial magnetic stimulation for unilateral spatial neglect in stroke patients. *J. Neurol. Sci.* 359 219–225. 10.1016/j.jns.2015.08.1541 26671118

[B49] Zhao X. Chen J. Chen M. C. Lv X. L. Jiang Y. H. Sun Y. H. (2014). Left-behind children in rural China experience higher levels of anxiety and poorer living conditions. *Acta Paediatri.* 103 665–670. 10.1111/apa.12602 24527673

