# Supplementary material for: Brain Structural Alterations in Left-Behind Children: A Magnetic Resonance Imaging Study
Source: Front Neural Circuits. 2019 May 8;13:33. doi: 10.3389/fncir.2019.00033 (PMC6517480; doi:10.3389/fncir.2019.00033)
Supplement: Supplementary file 1 [file Table_1.DOC]

**Supplementary Table 1. The mean FA value of each fiber tract for LBC and non-LBC participants.**

| Fiber tract (ROI) | Group | | Corrected |  |
| --- | --- | --- | --- | --- |
|  | LBC | Non-LBC | *P* | *t* |
|  | mean ± std | mean ± std |  |  |
| Middle cerebellar peduncle (MCP) | 0.44±0.02 | 0.43±0.02 | 0.59 | 0.54 |
| Pontine crossing tract (apart of MCP) | 0.45±0.02 | 0.45±0.03 | 0.30 | 1.04 |
| Genu of corpus callosum | 0.55±0.02 | 0.55±0.03 | 0.48 | 0.72 |
| Body of corpus callosum | 0.48±0.03 | 0.47±0.04 | 0.17 | 1.40 |
| Splenium of corpus callosum | 0.60±0.02 | 0.60±0.03 | 0.38 | 0.88 |
| Fornix (column and body of fornix) | 0.45±0.07 | 0.43±0.04 | 0.25 | 1.15 |
| Corticospinal tract R | 0.48±0.04 | 0.49±0.03 | 0.29 | 1.06 |
| Corticospinal tract L | 0.48±0.03 | 0.49±0.03 | 0.50 | 0.69 |
| Medial lemniscus R | 0.54±0.03 | 0.56±0.02 | <0.01* | 2.66 |
| Medial lemniscus L | 0.54±0.03 | 0.55±0.03 | 0.04 | 2.14 |
| Inferior cerebellar peduncle R | 0.43±0.03 | 0.44±0.03 | 0.36 | 0.92 |
| Inferior cerebellar peduncle L | 0.43±0.03 | 0.44±0.03 | 0.58 | 0.56 |
| Superior cerebellar peduncleR | 0.46±0.02 | 0.46±0.03 | 0.29 | 1.07 |
| Superior cerebellar peduncle L | 0.45±0.02 | 0.45±0.02 | 0.46 | 0.74 |
| Cerebral peduncle R | 0.61±0.02 | 0.60±0.03 | 0.59 | 0.54 |
| Cerebral peduncle L | 0.60±0.02 | 0.60±0.03 | 0.88 | 0.15 |
| Anterior limb of internal capsule R | 0.49±0.02 | 0.48±0.03 | 0.40 | 0.84 |
| Anterior limb of internal capsule L | 0.46±0.02 | 0.46±0.02 | 0.71 | 0.37 |
| Posterior limb of internal capsule R | 0.66±0.02 | 0.66±0.02 | 0.56 | 0.59 |
| Posterior limb of internal capsule L | 0.64±0.02 | 0.64±0.02 | 0.84 | 0.20 |
| Retrolenticular part of internal capsule R | 0.53±0.03 | 0.52±0.02 | 0.30 | 1.04 |
| Retrolenticular part of internal capsule L | 0.52±0.03 | 0.52±0.03 | 0.46 | 0.74 |
| Anterior corona radiate R | 0.40±0.02 | 0.39±0.03 | 0.44 | 0.78 |
| Anterior corona radiate L | 0.39±0.02 | 0.38±0.02 | 0.18 | 1.36 |
| Superior corona radiate R | 0.43±0.02 | 0.42±0.03 | 0.43 | 0.80 |
| Superior corona radiate L | 0.43±0.02 | 0.43±0.02 | 0.48 | 0.71 |
| Posterior corona radiate R | 0.41±0.02 | 0.41±0.02 | 0.73 | 0.35 |
| Posterior corona radiate L | 0.40±0.02 | 0.40±0.02 | 0.76 | 0.30 |
| Posterior thalamic radiation (include optic radiation) R | 0.51±0.02 | 0.51±0.03 | 0.78 | 0.27 |
| Posterior thalamic radiation (include optic radiation) L | 0.51±0.02 | 0.51±0.03 | 0.89 | 0.13 |
| Sagittal stratum (include inferior longitudinal fasciculus and inferior fronto-occipital fasciculus) R | 0.47±0.03 | 0.47±0.03 | 0.86 | 0.18 |
| Sagittal stratum (include inferior longitudinal fasciculus and inferior fronto-occipital fasciculus) L | 0.46±0.02 | 0.45±0.03 | 0.67 | 0.42 |
| External capsule R | 0.36±0.02 | 0.35±0.02 | 0.24 | 1.19 |
| External capsule L | 0.35±0.02 | 0.34±0.02 | 0.66 | 0.44 |
| Cingulum (cingulated gyrus) R | 0.32±0.02 | 0.31±0.03 | 0.08 | 1.77 |
| Cingulum (cingulated gyrus) L | 0.34±0.02 | 0.33±0.03 | 0.13 | 1.52 |
| Cingulum (hippocampus) R | 0.32±0.03 | 0.32±0.03 | 0.74 | 0.33 |
| Cingulum (hippocampus) L | 0.30±0.02 | 0.29±0.03 | 0.55 | 0.60 |
| Superior longitudinal fasciculus R | 0.38±0.02 | 0.37±0.03 | 0.12 | 1.58 |
| Superior longitudinal fasciculus L | 0.37±0.02 | 0.36±0.02 | 0.08 | 1.77 |
| Superior fronto-occipital fasciculus (could be a part of anterior internal capsule) R | 0.42±0.04 | 0.41±0.03 | 0.04* | 2.07 |
| Superior fronto-occipital fasciculus (could be a part of anterior internal capsule) L | 0.41±0.03 | 0.39±0.03 | 0.02* | 2.33 |
| Inferior fronto-occipital fasciculus R | 0.41±0.03 | 0.40±0.02 | 0.59 | 0.54 |
| Inferior fronto-occipital fasciculus L | 0.38±0.02 | 0.38±0.03 | 0.56 | 0.59 |
| Uncinate fasciculus R | 0.42±0.03 | 0.42±0.03 | 0.34 | 0.96 |
| Uncinate fasciculus L | 0.39±0.03 | 0.39±0.04 | 0.44 | 0.78 |
| Tapetum R | 0.41±0.03 | 0.42±0.03 | 0.75 | 0.32 |
| Tapetum L | 0.36±0.03 | 0.36±0.04 | 0.39 | 0.86 |

FA: fractional anisotropy; *p<0.05 corrected by multiple comparison correction with false discovery rate.
